# Yap1 alleviates sepsis associated encephalopathy by inhibiting hippocampus ferroptosis via maintaining mitochondrial dynamic homeostasis

**DOI:** 10.1111/jcmm.70156

**Published:** 2024-10-14

**Authors:** Xin Yang, Haifeng Duan, Sirui Li, Jing Zhang, Liang Dong, Jingli Ding, Xinyi Li

**Affiliations:** ^1^ Department of Anesthesiology Zhongnan Hospital of Wuhan University Wuhan Hubei China; ^2^ Hubei Provincial Engineering Research Center of Minimally Invasive Cardiovascular Surgery Wuhan China; ^3^ Wuhan Clinical Research Center for Minimally Invasive Treatment of Structural Heart Disease Wuhan China; ^4^ Department of Radiology Zhongnan Hospital of Wuhan University Wuhan Hubei China; ^5^ Liuzhou People's Hospital Affiliated to Guangxi Medical University Guangxi China; ^6^ Department of Gastroenterology Zhongnan Hospital of Wuhan University Wuhan Hubei China

**Keywords:** ferroptosis, mitochondrial homeostasis, sepsis, sepsis‐associated encephalopathy, Yap1

## Abstract

Sepsis‐associated encephalopathy (SAE) is a serious neurological complication accompanied by acute and long‐term cognitive dysfunction. Ferroptosis is a newly discovered type of cell death that is produced by iron‐dependent lipid peroxidation. As a key transcriptional coactivator in the Hippo signalling pathway, Yes‐associated protein 1 (YAP1) could target ferroptosis‐related genes. This study was aimed to determine whether Yap1 protects against SAE and inhibits ferroptosis via maintaining mitochondrial dynamic homeostasis. Caecal ligation puncture (CLP) was used to establish the SAE model, and LPS was applied in hippocampal cells to mimic the inflammatory model in vitro. The results showed that Yap1 conditional knockout in hippocampal caused lower survival in SAE mice and cognitive dysfunction, as proved by Morri's water maze (MWM) task, tail suspension test (TST), open field test (OFT) and elevated plus maze test (EPMT). After Yap1 knockout, the production of ROS, MDA and Fe^2+^ and proinflammatory cytokines in the hippocampus were increased, indicating that Yap1 deficiency exacerbates CLP‐induced brain injury and hippocampus ferroptosis. Meanwhile, GPX4, SLC7A11, ferritin (FTH1) and GSH levels were decreased in the Yap1 knockout group. In vitro, Yap1 overexpression mitigated LPS‐induced hippocampal cell ferroptosis and improved mitochondrial function by inhibiting mitochondrial fission, as evidenced by lower mitochondrial ROS, cell viability, Fe^2+^ and the expression of Fis1 and Drp1. Further, the present study suggested that Yap1 could inhibit ferritinophagy‐mediated ferroptosis in the hippocampus via inhibiting mitochondrial fission, thus reducing cognitive dysfunction in SAE mice.

## 1 INTRODUCTION

Sepsis is a multi‐organ dysfunction caused by infection, which is a significant cause of adult ICU mortality.^1^ The most common and serious sepsis neurological complication is sepsis‐associated encephalopathy (SAE), which is marked by changes in cognitive function and state of consciousness.^2^, ^3^ With the high incidence of SAE, it is a risk factor that affects the prognosis of sepsis patients independently.^4^ Recent studies have indicated that brain damage occurs in 9%–71% of sepsis patients, and these patients tend to have a higher mortality rate.^5^ Long‐term impairment of cognition and altered mental state can persist for years even in people who survive SAE.^6^ Emerging evidence has suggested that neuroinflammation, apoptosis and neurotoxicity are associated with SAE; however, the mechanisms of SAE are complicated. Elucidating the underlying molecular mechanisms of SAE will be significant for discovering new therapies.

Recently, the role of mitochondria in SAE has attracted much attention. Mitochondrial oxidative phosphorylation in eukaryotic cells is the primary site for ATP production, accompanied by continuous fusion, fission occurring within mitochondria, forming a highly interconnected network of dynamic tubular in the energy metabolism of neurons. Studies over the past decade indicated dysfunctional mitochondrial dynamic network is a key pathological mechanism in central nervous system diseases.^7^ According to recent research, the first stage of SAE is thought to be an energy metabolism disorder of neurons^8^ and the dysfunction caused by abnormal mitochondrial fusion, division and transport is linked to the pathogenesis of SAE.^9^

Ferroptosis is an iron‐dependent kind of programmed cell death distinct from necrosis, apoptosis and autophagy.^10^ Biochemically, iron overload, inactivation of glutathione peroxidase 4 (GPX4), glutathione (GSH) depletion and lipid peroxidation are the key factors resulting in ferroptosis.^11^ Ferritinophagy is a process of autophagic degradation of ferritin heavy chain 1 (FTH1) mediated by nuclear receptor coactivator 4 (NCOA4). During the process of ferritinophagy, reactive oxygen species (ROS) in large amounts are released, resulting in inferroptosis. Ferroptosis is characterized by ultrastructural changes in mitochondria, and recent research suggests that mitochondrial damage is a major contributor to ferroptosis.^12^ Mitochondrial damage has been related to ferroptosis in diseases such as cancer and cardiovascular disease.^13^, ^14^ Lin Q revealed the important effect of mitochondria on ferroptosis.^15^ Dixon SJ showed that ferroptosis led to smaller mitochondria with elevated mitochondrial membrane density.^16^ Basit F reported that ferroptosis inhibitors can rescue cell toxicity induced by inhibiting mitochondrial complex I.^17^ It is generally accepted that the maintenance of mitochondrial homeostasis significantly correlates with ferroptosis resistance.

The Hippo/Yap signalling pathway is highly conserved in mammals and regulates cell differentiation, proliferation and apoptosis.^18^ Yap1 is a transcriptional coactivator that is suppressed by the Hippo signalling pathway. Large tumour suppressor 1/2 (LATS1/2) can phosphorylate and inactivate Yap1, resulting in Yap1 retention in the cytoplasm.^19^ Studies over the past decade indicate that the Yap1 plays key roles in modulating mitochondrial function and ferroptosis in various diseases. Banerjee demonstrated that Yap1 overexpression increased the number of mitochondria and enhanced mitochondrial function, which implied a possible connection between Yap and mitochondrial function.^20^ In addition, Sun X revealed a key role of Yap1 in cardiomyocyte mitochondrial oxidative damage and ferroptosis.^21^ Recent researches suggest that the Hippo/Yap signalling pathway is important for the development of the central nervous system.^22^, ^23^ After spinal cord injury, Yap1 was upregulated and Hippo kinase‐dependent selective activation in astrocytes.^24^ The mitochondrial function in Yap1 knockout mice is impaired, whereas Yap1 can reduce nerve inflammation and inhibit microglial cell activation after acute cerebral ischemia reperfusion injury.^25^ The possible mechanism of Yap1 in ferroptosis during SAE remains unknown. As a result, this research intends to illustrate the function of Yap1 in ferroptosis in in vivo and in vitro models of SAE.

## 2 MATERIALS AND METHODS

### 2.1 Animals

We got adult male C57BL/6 mice from Cyagen in Suzhou, China. YAP1^flox/flox^ mice were hybridized with Pomc‐CreERT2 mice to produce hippocampus‐specific YAP1‐conditional knockout mice (termed as YAP1^f/f^). Mice aged 6–8 weeks were intraperitoneally injected with tamoxifen (dissolved in maize oil) at a dose of 75 mg/kg once a day (75 mg/kg, once a day) for 5 consecutive days, followed by a 4 week rest period, to create hippocampus‐specific YAP1 conditional knockout mice. In this way, YAP1‐conditional knockout animals were successfully obtained. YAP1^flox/flox^ mice were used as wild type (WT) for control. Under a 12‐h light/dark cycle, all animals had unfettered access to sustenance and water. All animals were accustomed to the environment for at least 1 week before any experiments. We followed the National Institutes of Health Guide for the Care and Use of Laboratory Animals. The study was approved by the Ethical Committee for Animal Research of Zhongnan Hospital affiliated to Wuhan University (Approval no.: ZN2021187).

### 2.2 Caecal ligation and puncture (CLP) model

CLP was used to generate the sepsis model. WT mice and YAP1‐knockout mice were divided into four groups at random: sham operation (WT+ Sham), CLP model (WT + CLP), Yap1‐conditional knockout sham operation (Yap1^f/f^ + Sham) and Yap1‐conditional knockout CLP model (Yap1^f/f^ + CLP). CLP was applied to the WT + CLP and Yap1^f/f^ + CLP groups. Before surgical procedure, pentobarbital sodium 50 mg/kg was used to anaesthetize mice. A heating pad (Physitemp Instruments Inc., Clifton, NJ, USA) was used to preserve body heat. At the middle of the lower abdomen, a 1‐cm incision was made, and the cecum was dissected, ligated and pierced twice with an 18‐gauge needle. Following that, the cecum was repaired and the belly cavity was sutured after the faeces were extracted through the puncture wound. The animals were infiltrated with 0.25% bupivacaine at the operative site. For the sham group, the cecum was simply rolled over and the abdomen was instantly closed.

### 2.3 Morris water maze (MWM) task

MWM test was used to estimate spatial learning and memory function.^26^ For the MWM task, mice received the training in a white circular pool with a 25 cm^2^ concealed platform 1 cm below the water. Before each trial, the mice were put on the platform for 20 s to orientate additional maze cues. Then mice were gently put into the pool from one of the three places, facing the wall and the time spent to the platform was recorded. During the probe trial, the platform was removed and mice were freed from their initial position. The swimming distance, swimming time in the target quadrant and frequency of crossing the platform were recorded during the 60‐s memory test.

### 2.4 Elevated plus maze test (EPMT)

The 40 cm high plus maze consisted of a central square with two closed arms and two open arms. The open arms were 40 cm in length and 10 cm in width, with no walls on either side, whereas the closed arms were surrounded on both sides by 10 cm high walls. The four arms stretched from a center square for 10 cm diameter. Facing one of the open limbs, mice were placed in the center square during the experiment. They have 5 min to examine all of the arms. The arms were washed with 75% ethanol after one mouse completed the session. Time spent in each of two open arms was calculated. Entries were defined as four mouse paws included in an arm.

### 2.5 Tail suspension test (TST)

The test was carried out with some modifications according to a previous study.^27^ The adhesive tape was used to secure the mouse's tail to a 50 cm tall holder. The procedure took 5 min. For data analysis, mice that appeared to be at rest for the first time were recorded, and the total immobility time during the 5 min was calculated.

### 2.6 Open field test (OFT)

The OFT was carried out in a box (50 × 50 cm) with four transparent walls of 50 cm height. The mice were placed individually in the centre of the box and had access to move freely for 5 min. 75% ethanol was used to clean the box after each trial. On average, the bottom of the box was divided into 25 grids, with the 9 grids in the center defining the central area. A tracking analysis system was used to record the travel path within 5 min (Coulbourn Instruments, Holliston, MA). The time spent in centre was calculated to evaluate extent of anxiety.

### 2.7 HE staining

The tissue was immersed in 4% paraformaldehyde and dehydrated in 95% alcohol before being cleared and embedded in paraffin. After being divided into 8 mm dry pieces, the tissue was rehydrated using a series of alcohols. Contamination was avoided during staining, and sections were kept dry. A light microscope was used to examine histopathological changes. The hippocampal regions of each mouse were chosen at random, and the neurons were counted using ImageJ.

### 2.8 Electron microscopic analysis

The tissues were removed and immersed with 4% paraformaldehyde and 1% glutaraldehyde. The brains were then dehydrated in a series of ethanol concentrations before being sectioned into ultrathin layers. Following this, the segments were stained with uranyl acetate and Reynold's lead citrate. Using a Philips CM 10 electron microscope (Hitachi Co., Tokyo, Japan), the sections were examined. Six sections were randomly observed in each group and analysed.

### 2.9 Nissl's staining

The brain tissues were routinely exposed to water as follows: xylene I and xylene II for 15 min each. After that, they were dehydrated with a range of alcohol: 100%I, 100%II, 95%, 90%, 80%, 70% and 50% for 5 min each. Finally, they were washed three times with distilled water for 5 min each. The tissues were then treated for 40 min at 60°C with 1% toluidine blue. The dye brains were washed with distilled water, then dried in 70%, 80%, 95% and 100% ethanol, transparent with xylene, and then sealed with neutral gum. A microscope was used to observe and take images of the neurons.

### 2.10 Enzyme‐linked immunosorbent assay (ELISA)

The tissue of hippocampus was dissected and homogenized. After 10 min of centrifuging at 15,000 × *g*, we collected the supernatants. The supernatants of culture medium were collected for cytology experiment. ELISA assay kits were used to examine tumour necrosis factor (TNF)‐α (cat. no. E‐EL‐M0049c), IL‐6 (cat. no. E‐EL‐M0044c), and IL‐1β (cat. no. E‐EL‐M0046c). The steps were done following the manufacturer's guidelines.

### 2.11 Western blotting

Brain tissues were collected at predetermined intervals and homogenized with an ice‐cold RIPA lysate buffer (Beyotime, Beijing, China), which contained multiple protease inhibitors (Merck Millipore, MA, USA). The samples were then lysed for 30 min on ice and centrifuged at 4°C with 12,000 × *g* for 10 min at and. The concentrations of protein were ascertained using Bicinchoninic Acid Protein Assay Kit (Beyotime, Beijing, China). Following separation on 8%–12% sodium dodecyl sulphate–polyacrylamide (SDS–PAGE) gels, protein samples (10 g/lane) were electroblotted onto polyvinylidene fluoride (PVDF) membranes. GPX4, Yap1, FIN1, NCOA4, SLC7A11, Mfn1, Opa1, Drip1, Fis1 and β‐actin were incubated overnight at 4°C on PVDF membranes, which blocked in TBST buffer with 5% nonfat dried milk. Peroxidase‐conjugated goat anti‐rabbit or anti‐mouse immunoglobulin G (IgG) antibodies (ZSBIO, Beijing, China) were incubated at 37°C for an hour with the membranes. The PVDF membrane was rinsed with TBST three times and incubated for 2 h at room temperature with secondary antibodies conjugated with horseradish peroxidase (1:5000, Abcam, Cambridge, UK). After three times TBST rinses, the bands were detected using enhanced chemiluminescence (ECL, Themo Fisher China, Shanghai, China) and X‐ray films. ImageJ (National Institutes of Health, Bethesda, MD) was used to quantify the relative levels of the target protein.

### 2.12 Immunohistochemical stain (IHC stain)

The tissues were collected and postfixed in 4% PFA at 4°C for 8 h, then dehydrated overnight in 30% sucrose at 4°C. The brain slices were immersed for 30 min in 0.5% Triton X‐100 for permeation, followed by an hour of blocking in 3% bovine serum albumin at room temperature. The segments were immersed with anti‐NCOA4 and anti‐FTH1 antibodies at 4°C overnight. The cells were washed with PBS three times before being treated with secondary antibodies for 2 h at normal temperature. After PBS rinsing, segments were stained with DAPI. Pictures were taken by the Olympus confocal microscope and images were analysed with ImageJ software.

### 2.13 Establishment of cell models

The HT22 cell line (hippocampal neuron cells) was used for in vitro experiments. The cells were cultured in minimum Eagle's medium (MEM) with 10% foetal bovine serum (DMEM, Gibco, Grand Island, USA) and 1% streptomycin and penicillin (both from Invitrogen; Thermo Fisher Scientific, Inc., Waltham, MA, USA). They were kept at 37°C with 5% CO_2_ and a Forma 3 (Thermo Fisher Scientific, Inc.).

### 2.14 Cell transfection

HT22 cells were transfected with Yap1 overexpression (Yap1 OE) clone lentiviral particle (supplied by Genechem Co. Ltd., Shanghai, China) or siRNA specific for Yap1 by using Lipofectamine® 2000 transfection reagent (supplied by Genechem Co. Ltd., Shanghai, China). The transfection procedure was conducted following the manufacturer's guides. Stable Yap1 OE and Yap^f/f^ HT22 cells were selected to for detection after 48 h culture. Then, cells were cocultured with the indicated reagents and LPS (1 g/mL) for 24 h.

### 2.15 Cell viability assay and MTT assays

The cell viability was determined by the Cell Counting Kit‐8 (CCK‐8) assay kit (Beyotime, Shanghai, China). Cells were loaded with a 5 μM JC‐1 dilution at 37°C in dark for 30 min. The cells were observed using fluorescence microscope (TE‐2000, Nikon Co., Tokyo, Japan).

### 2.16 Detection of ROS levels

A dihydroethidium (DHE) fluorescent probe (D7008, Sigma‐Aldrich, MO, USA) was used to measure the level of reactive oxygen species (ROS) in brain tissue. Briefly, frozen brain tissues were incubated in 50 μM of DHE at room temperature and away from light for 1 h, and then incubated with DAPI for 10 min. With the help of fluorescence microscope (TE‐2000, Nikon Co., Tokyo, Japan), the fluorescence intensity at 525 nm excitation (Ex) and 610 nm emission (Em) wavelengths was measured.

HT22 cells were cultured in 0.25% trypsin. Then HT22 cells were centrifuged at 1500 rpm under 4°C for 5 min. Then they were incubated with 2′,7′‐dichlorofluorescein diacetate (DCFH‐DA) fluorescent probe (D6883, Sigma‐Aldrich, MO, USA). After 1 h of dark incubation with DCFH‐DA, DAPI treated cells for 10 min. Fluorescence microscope (TE‐2000, Nikon, Co., Tokyo, Japan) was used to test ROS levels in HT22 cells. Using flow cytometry (BD Accouri C6 plus, BD Biosciences, USA) and Flow J software with the BODIPYTM 581/591 C11 (D3861, Invitrogen) fluorescent probe in HT22 cells, the lipid ROS levels were measured.

### 2.17 Detection of mitochondrial ROS

HT22 cells were seeded in a 24‐well Luci fugal plate and cultured for 10 min with MitoSOX™ reagent solution. They were washed twice with PBS after that. Using a fluorescence microscope (Nikon, Co., Tokyo, Japan), the level of mitochondrial ROS was measured.

### 2.18 Ferritin and LAMP2

HT22 cells were cultured in medium minus, then immunostained with anti‐ferritin (red) and anti‐LAMP2 (green) antibodies. Using confocal laser scanning microscopy and fluorescence microscopy (TE‐2000, Nikon, Co., Tokyo, Japan), HT22 cells were photographed.

### 2.19 Co‐immunoprecipitation (IP) assay

Using BCA protein assay kit (Beyotime, Shanghai, China), the concentration of protein was detected in HT22 cells. Immunoprecipitation was conducted using 1 mg total protein lysates extracted from each group (IP). The rabbit polyclonal IgG control antibody was used to hatch the specimen. After 4 h of rotation at 4°C, a total volume of 25 μL of resuspended protein A/G plus agarose was mixed with the lysates, and rotated for two more hours. After washing and denaturizing the immunoprecipitation buffer, eluted proteins were immunoblotted with antibodies (anti‐NCOA4, anti‐Ferritin) for the co‐IP experiment.

### 2.20 Determination of related indicators

The mRNA sequence of Yap1 gene provided by GenBank was used to design and synthesize interference sequences, and the recombinant lentiviral vector was transfected into HT22 cells to create Yap1 gene‐silenced cells.

Using MDA assay kit (Beyotime, China), GSH assay kit (Beyotime, China), and iron assay kit (MAK025, Sigma‐Aldrich, MO, USA), the levels of glutathione (GSH), malondialdehyde (MDA) and Fe^2+^ in tissue lysates and HT22 cells were measured.

### 2.21 Statistical analysis

The data were presented as mean ± SEM and analysed by SPSS 20.0. Independent *t*‐test and one‐way analysis of variance (ANOVA) were used to analyse the differences between groups. Dunnett's test was used for multiple comparisons correction. When comparing multiple groups, data were analysed using two‐factor ANOVA followed by Tukey's post hoc test when appropriate. Kaplan–Meier survival curves and log‐rank analyses were used to compare survival outcomes between different groups. *p* < 0.05 was considered statistical significance.

## 3 RESULTS

### 3.1 Knockout of Yap1 decreased survival rate and aggravated cognitive and emotional dysfunction of CLP‐induced sepsis in mice

**FIGURE 1:**
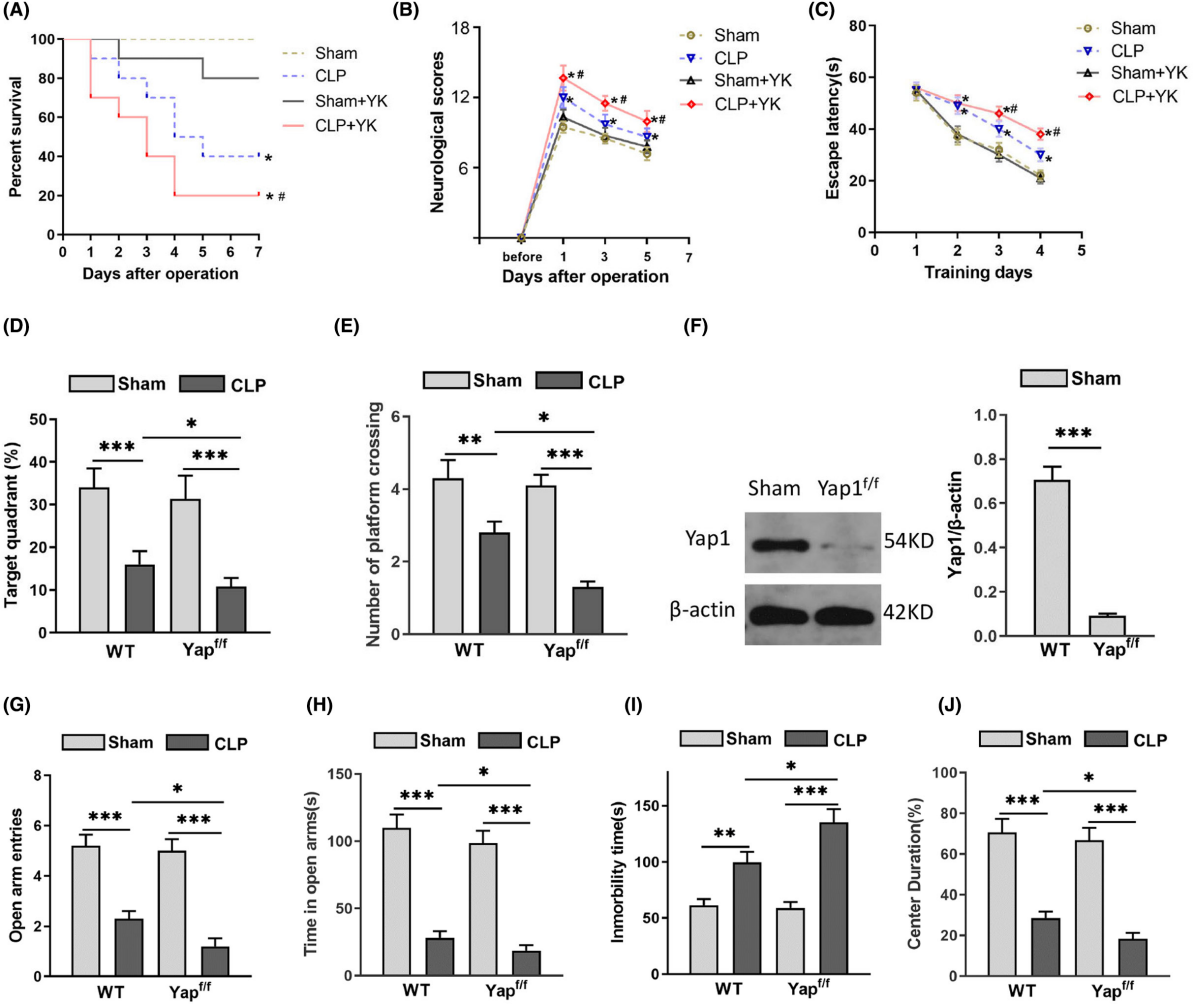
Yes‐associated protein 1 (Yap1) deficiency aggravated sepsis‐induced cognitive dysfunction and emotional of in caecal ligation puncture (CLP) mice. (A) The survival rate of each group after CLP. (B) The neurological score before and after CLP procedure in each group. (C) The escape latency of mice during the training days in MWM. (D, E) In MWM, the percentage of time spent in the quadrant and the times of crossing the platform in postoperative probe test of each group. (F) The certification and efficiency of Yap1 conditional knockout in mice hippocampus. (G, H) In elevated plus maze test (EPMT), the open arm entries of mice and time spent in the open arms. (I) The immobility time of mice in tail suspension test (TST). (J) The time spent in the center area in open field test (OFT). Data are presented as mean ± SEM. *p < 0.05, **p < 0.01, ***p < 0.001 and ^#^ *p* < 0.05 (*N* = 8–12 mice per group).

A CLP mouse model was established, and MWM was conducted to detect the spatial learning and memory ability after CLP. There was no difference in survival rate and neurological function score among groups before surgery. The survival rate was 100% in each group before surgery, however, it decreased with the time in the WT + CLP group, the Yap1^f/f^ + Sham group and Yap1^f/f^ + CLP group after surgery (Figure 1A). The survival rate in CLP groups decreased, indicating successful establishment of CLP model, while the neurological function score of the CLP group increased compared with the sham group (Figure 1B). Furthermore, Yap1 deletion caused higher neurological function scores. During the training days, the CLP mice had a longer escape latency than sham mice, and Yap1^f/f^ mice performed worse than wild‐type mice. They had longer escape latency (Figure 1C). In postoperative probe test, the percentage of time spent in the target quadrant and the times of crossing the platform were significantly lower in CLP mice than that in sham group, and Yap1^f/f^ mice spent less time and had fewer crossing times than wild‐type mice after CLP (Figure 1D; (interaction: CLP× Yap^f/f^, *F* = 7.133, *p* = 0.0054; CLP: *F* = 14.206, *p* < 0.0001; Yap^f/f^: *F* = 3.537, *p* = 0.0342) and E; (interaction: CLP× Yap^f/f^, *F* = 8.275, *p* = 0.0036; CLP: *F* = 12.852, *p* < 0.0001; Yap^f/f^: *F* = 4.294, *p* = 0.0258)) These results indicated that sepsis may cause cognitive impairment in mice, while Yap1 knockout could aggravate learning and memory impairment caused by sepsis. The knockout efficiency of Yap1 was tested by western blot. The expression of Yap1 in the hippocampus was decreased in the Yap1^f/f^ mice compared with wild‐type mice (Figure 1F, *p* < 0.001).

After CLP, EPMT, OFT and TST were carried out to investigate the effects of Yap1 on the emotional behaviour of mice. In the EPMT, the entries and time spent in the open arms of the CLP group were less than those of the sham group, and the entries and duration time in the open arms of septic mice after CLP were less than that of the wild‐type mice (Figure 1G; (interaction: CLP× Yap^f/f^, *F* = 8.259, *p* = 0.0034; CLP: *F* = 16.245, *p* < 0.0001; Yap^f/f^: *F* = 4.368, *p* = 0.0243) and H (interaction: CLP× Yap^f/f^, *F* = 10.212, *p* = 0.0001; CLP: *F* = 14.206, *p* < 0.0001; Yap^f/f^: *F* = 3.124, *p* = 0.0423)). The immobility time of CLP mice increased in TST compared to sham group, and Yap1^f/f^ mice had longer immobility time than wild‐type mice after CLP (Figure 1I; (interaction: CLP× Yap^f/f^, *F* = 9.366, *p* = 0.0022; CLP: *F* = 13.012, *p* < 0.0001; Yap^f/f^: *F* = 4.012, *p* = 0.0122)). In OFT, mice spent less time in the central area in the CLP group, and even less time after Yap1 conditional knockout (Figure 1J (interaction: CLP× Yap^f/f^, *F* = 8.584, *p* = 0.0029; CLP: *F* = 12.542, *p* < 0.0001; Yap^f/f^: *F* = 2.984, *p* = 0.0436)), indicating that sepsis could cause emotional disorders in mice, and that Yap1 knockout could exacerbate these behavioural deficits.

### 3.2 Yap1 deficiency exacerbated sepsis‐induced hippocampus ferroptosis and mitochondrial fission in CLP mice

**FIGURE 2:**
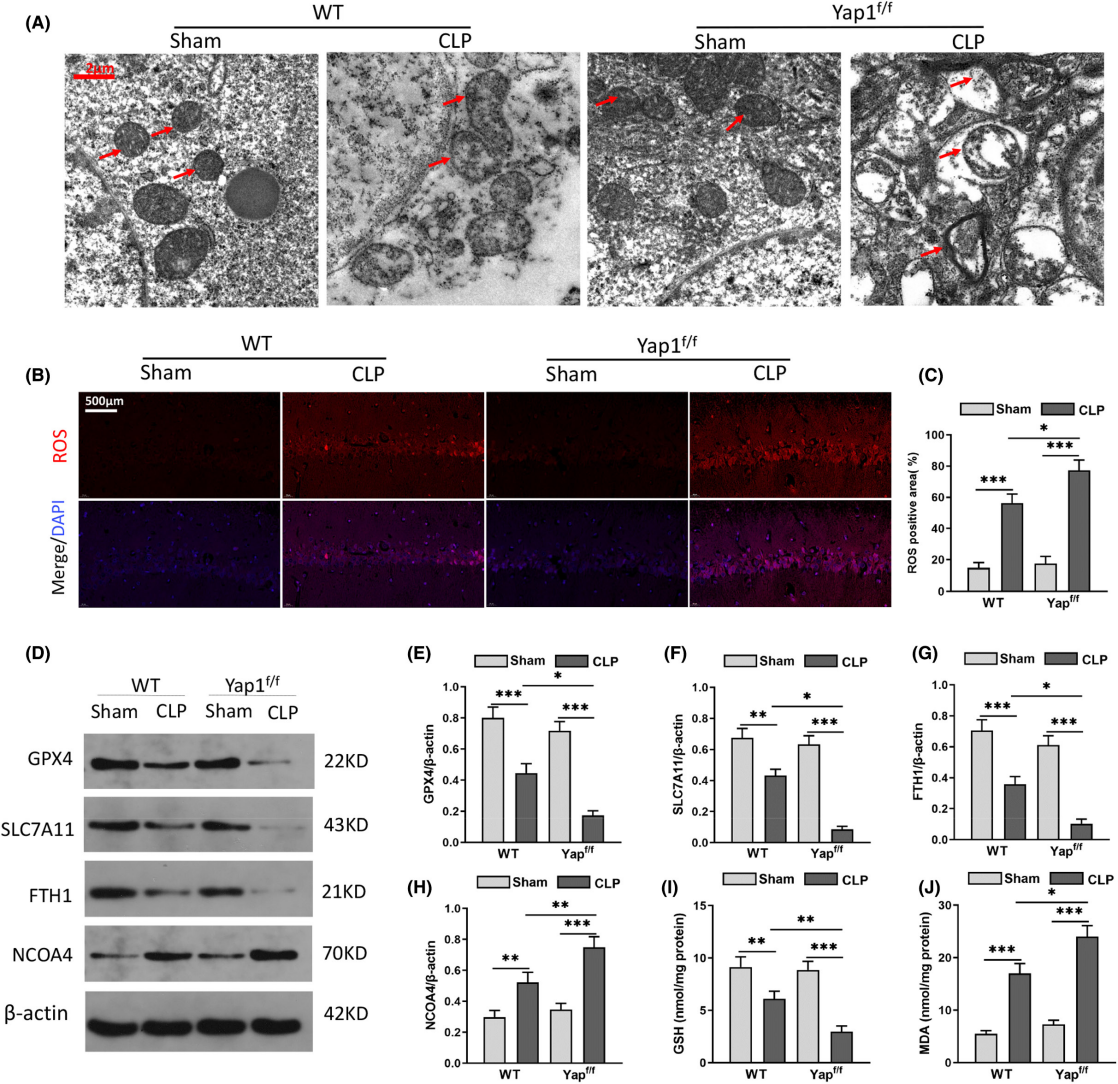
Yes‐associated protein 1 (Yap1) knockout exacerbated CLP‐induced ferroptosis and mitochondrial fission of hippocampus in caecal ligation puncture (CLP) mice. (A) Representative images captured by TEM. The red arrow represents representative mitochondria in hippocampus of wild type (WT) and Yap1^f/f^ mice treated by CLP or not. (B, C) The fluorescent staining images of ROS and the quantification of ROS fluorescence intensity in hippocampus of each group. (D) Western blot showed the expression of GPX4, SLC7A11, FTH1, NCOA4 protein in hippocampus. (E–H) Quantitative results of GPX4, SLC7A11, FTH1 and NCOA4 in mice hippocampus of different group. (I, J) The content of GSH and MDA in hippocampus detected by commercial kit. Data are presented as mean ± SEM. **p < 0.01, ***p < 0.001 and ^#^ *p* < 0.05 (*N* = 8–12 mice per group).

TEM had captured the morphological characteristics of ferroptosis and mitochondrial fission. The results revealed CLP induced aberrant mitochondrial (red arrows), including hippocampal mitochondria vacuolation and more mitochondrial fragments, and the mitochondrial crest was fuzzy and unclear. The morphology and volume of hippocampal mitochondria in the sham group were normal, and most of them were oval and columnar, with an obvious mitochondrial crest. Moreover, YAP1 deficiency exacerbated mitochondrial injury caused by CLP (Figure 2A). Ferroptosis is characterized by iron‐dependent lipid peroxidation. The ROS level and the expression of GPX4, SLC7A11, FTH1, NCOA4, GSH and MDA in the hippocampus were determined to investigate the effect of Yap1 in ferroptosis and mitochondrial fission on SAE. The data indicated that the content of ROS increased in CLP mice and knockout of Yap1 elevated the production of ROS (Figure 2B and C; interaction: CLP× Yap^f/f^, *F* = 13.421, *p* < 0.0001; CLP: *F* = 16.052, *p* < 0.0001; Yap^f/f^: *F* = 3.234, *p* = 0.0321). The expression of NCOA4 and MDA were upregulated, while GPX4, SLC7A11, FTH1 and GSH were downregulated in CLP mice compared with that in sham group (Figure 2 D–J; (E: interaction: CLP× Yap^f/f^, *F* = 6.548, *p* = 0.0032; CLP: *F* = 18.254, *p* < 0.0001; Yap^f/f^: *F* = 2.358, *p* = 0.0421); (*F*: interaction: CLP× Yap^f/f^, *F* = 5.857, *p* = 0.0045; CLP: *F* = 15.621, *p* < 0.0001; Yap^f/f^: *F* = 3.125, *p* = 0.0398); (G: interaction: CLP× Yap^f/f^, *F* = 6.245, *p* = 0.0037; CLP: *F* = 13.245, *p* < 0.0001; Yap^f/f^: *F* = 2.892, *p* = 0.0457); (H: interaction: CLP× Yap^f/f^, *F* = 9.214, *p* < 0.0001; CLP: *F* = 19.725, *p* < 0.0001; Yap^f/f^: *F* = 7.895, *p* = 0.0017); (*I*: interaction: CLP× Yap^f/f^, *F* = 8.51, *p* = 0.0024; CLP: *F* = 18.952, *p* < 0.0001; Yap^f/f^: *F* = 6.212, *p* = 0.0008); (J: interaction: CLP× Yap^f/f^, *F* = 5.986, *p* = 0.0048; CLP: *F* = 15.301, *p* < 0.0001; Yap^f/f^: *F* = 3.264, *p* = 0.0403)). Besides, compared to wild‐type, knockout of Yap1 increased ROS, NCOA4 and MDA expression and decreased the content of GPX4, SLC7A11, FTH1 and GSH after CLP (Figure 2D–J). Collectively, these data indicated that knockout of Yap1 in mice deteriorated ferroptosis and mitochondrial fission in the hippocampus after CLP stimulation.

### 3.3 Yap1 knockout aggravated CLP‐induced microglial activation and neuroinflammation via ferroptosis resistance

**FIGURE 3:**
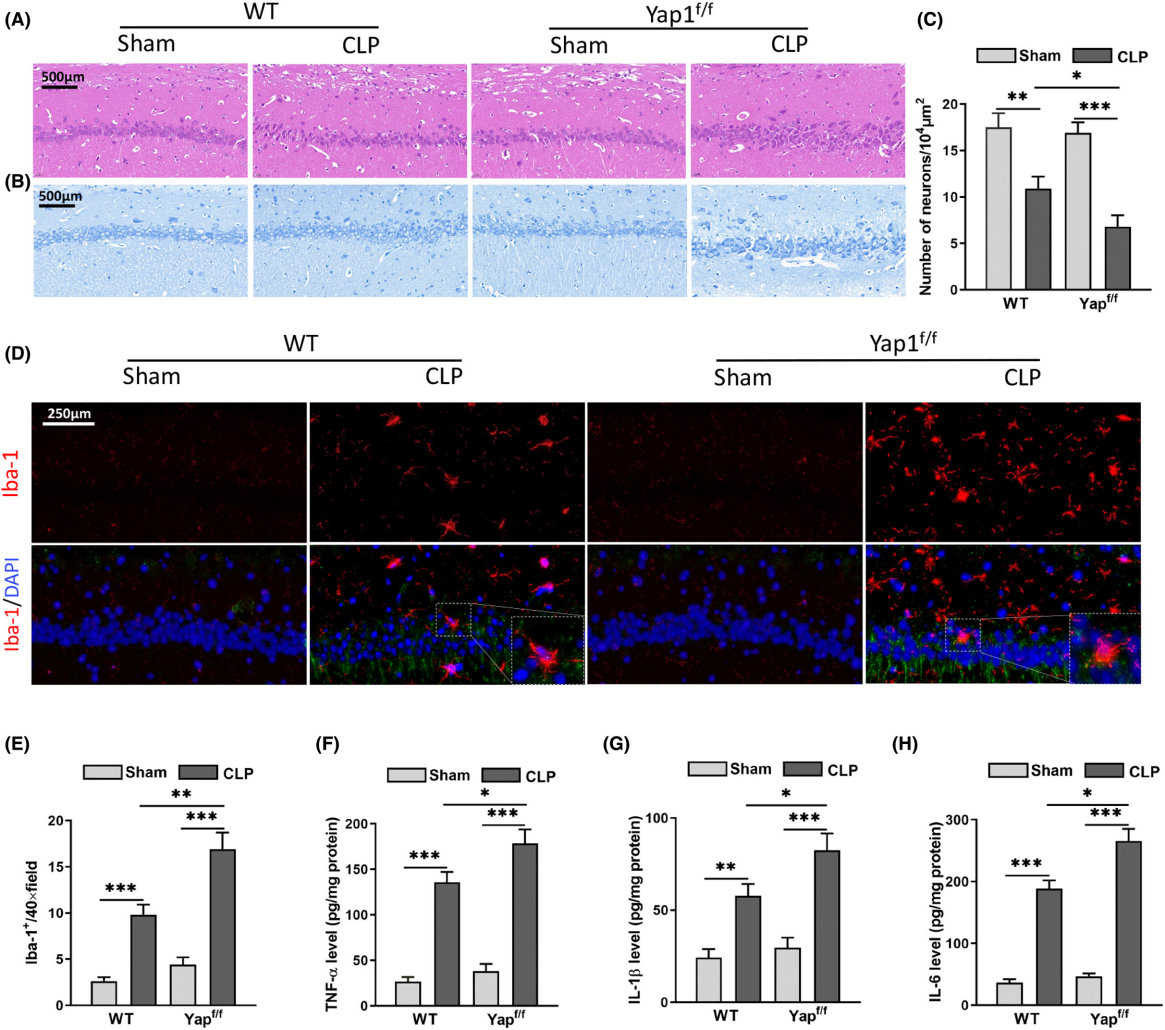
Yes‐associated protein 1 (Yap1) knockout aggravated caecal ligation puncture (CLP)‐induced microglial activation and neuroinflammation via ferroptosis resistance. (A, B) HE and Nissl staining images of hippocampal CA1 region in mice following CLP or YAP1^f/f^ treatment or both. (C) The number of surviving neurons in hippocampal CA1 region. (D, E) The microglia stained by immunofluorescence with Iba‐1. (F–H) The content of TNF‐α, IL‐1β and IL‐6 detected by ELISA assay kits. Data are expressed as mean ± SEM. **p < 0.01, ***p < 0.001 and ^#^ *p* < 0.05 (*N* = 8–12 mice per group).

Because spontaneous locomotor activity is related to the densities and functions of neurons in CA1 of the hippocampus, we examined whether CA1 neuronal injury contributes to such behavioural deficits. Yap1 conditional knockout mice were used to confirm the role of Yap1‐related neuronal injury in sepsis‐induced SAE in vivo. After CLP, neuronal damage was examined using H&E and Nissl stains. CLP caused significant pathological changes, including hippocampal CA1 neuronal loss, as demonstrated by a significant decrease in Nissl‐stained cells and massive inflammatory cell infiltration, while the number of surviving neurons was higher in CLP wild‐type mice than in CLP Yap1^f/f^ mice Figure 3A–C; F: interaction: CLP× Yap^f/f^, (*F* = 6.986, *p* = 0.0074; CLP: *F* = 15.243, *p* < 0.0001; Yap^f/f^: *F* = 4.236, *p* = 0.0095). These findings confirmed the importance of Yap1 in CLP‐induced brain injury.

To confirm that Yap1 exacerbates CLP‐induced microglial activation and neuroinflammation, microglia in wild‐type and Yap1^f/f^ mice were detected with Iba‐1 by immunofluorescent staining. Iba‐1 is a marker of activated microglia. The inflammatory response is reflected in microglial polarization. Immunofluorescence staining revealed that the CLP group contained more Iba1^+^ microglia than the sham group. CLP increased the number of Iba1^+^ hippocampus cells in both wild‐type and Yap1^f/f^ mice, and Yap1 knockout led to an increase in the number of Iba1^+^ cells (Figure 3D,E; F: interaction: CLP× Yap^f/f^, *F* = 8.756, *p* = 0.0006; CLP: *F* = 19.245, *p* < 0.0001; Yap^f/f^: *F* = 6.245, *p* = 0.0038), demonstrating that Yap1 alleviated neuroinflammation by switching microglia polarization.

Using ELISA test kits, inflammatory cytokines were discovered in the hippocampus to explore the inflammatory microenvironment during sepsis. TNF‐α, IL‐1 and IL‐6 levels were increased in the CLP group than the sham group (Figure 3F–H; (*F*: interaction: CLP× Yap^f/f^, *F* = 7.584, *p* = 0.0048; CLP: *F* = 15.684, *p* < 0.0001; Yap^f/f^: *F* = 3.954, *p* = 0.0198); (G: interaction: CLP× Yap^f/f^, *F* = 8.214, *p* = 0.0043; CLP: *F* = 12.654, *p* < 0.0001; Yap^f/f^: *F* = 4.526, *p* = 0.0215); (H: interaction: CLP× Yap^f/f^, *F* = 6.954, *p* = 0.0068; CLP: *F* = 15.214, *p* < 0.0001; Yap^f/f^: *F* = 4.102, *p* = 0.0301)), indicating that inflammatory factors induced by sepsis infiltrate the hippocampus. Yap1 knockout significantly increased TNF‐α, IL‐1 and IL‐6 levels in the CLP group (Figure 3F–H), indicating that Yap1 could make changes to the inflammatory microenvironment by decreasing pro‐inflammatory cytokines.

### 3.4 Yap1 overexpression improved LPS‐induced mitochondrial dysfunction via modulating mitochondrial dynamic homeostasis in vitro

**FIGURE 4:**
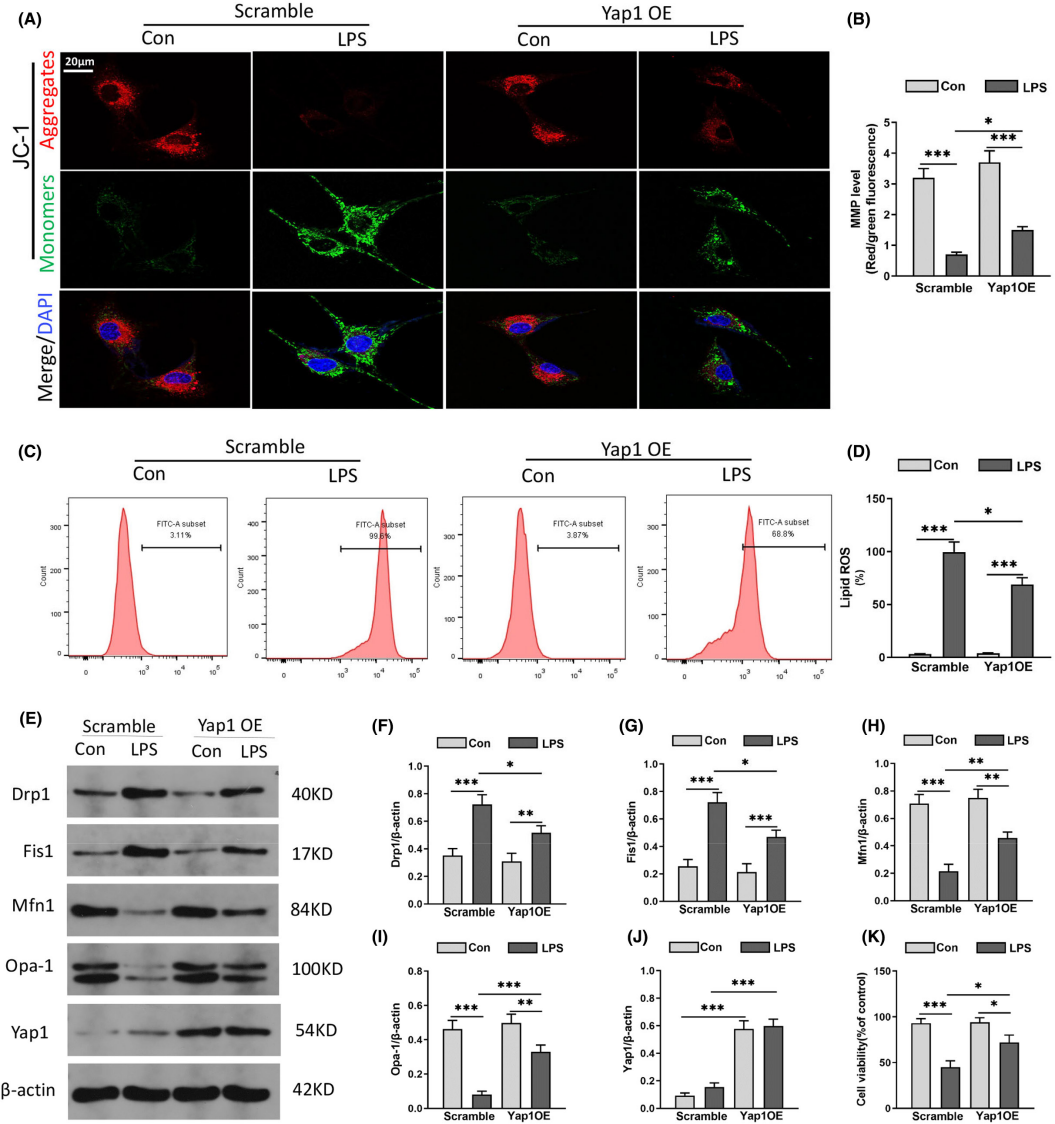
Yes‐associated protein 1 (Yap1) overexpression improved LPS‐induced mitochondrial dysfunction via modulating mitochondrial dynamic homeostasis in vitro. (A, B) The mitochondrial membrane potential (MMP) was visualized by JC‐1 in HT22 cells. (C, D) Lipid ROS production was determined by flow cytometry in HT‐22 cells and the quantification of lipid ROS. (E) Western blot showed the expression of Drp1, Fis1, Mfn1 and Opa‐1 protein in HT22 cells. (F–I) The relative quantification of Drp1, Fis1, Mfn1 and Opa‐1 expression. (J) The certification and efficiency of YAP1 conditional knockout in HT22 cells. (K) Cell viability was evaluated by the Cell Counting Kit‐8. Data are expressed as mean ± SEM. *p < 0.05, **p < 0.01, ***p < 0.001.

To explore the mitochondrial homeostasis in LPS‐induced hippocampus injury, HT22 cells were transfected with Yap1 overexpressing lentivirus (Yap1 OE). Mitochondrial membrane potential (MMP) is closely related to the normal function of mitochondria, and JC‐1 is a specific mitochondrial dye method that reflects MMP according to the changes in fluorescence. After LPS, the intensity of green fluorescent increased while the intensity of red fluorescent decreased, indicating a decreased MMP. While Yap1 overexpressed increased the number of green fluorescent mitochondria and decreased red fluorescent mitochondria (Figure 4A,B), suggesting Yap1 overexpression could be elevated.

Flow cytometry revealed a significant increase in mitochondrial ROS production after LPS, in Figure 4C,D, the production of mitochondrial ROS was increased, indicating increased mitochondrial oxidative stress. The data showed that ROS was increased after LPS. Furthermore, the production of ROS was inhibited after the overexpression of the Yap1 (Figure 4C,D). In order to verify the efficiency of lentivirus, we determined the expression of Yap1 protein. The results revealed that Yap1 levels decreased after LPS, while it significantly increased in the LPS group after Yap1 overexpression (Figure 4E,J). The expression of Drp1, Fis1, Mfn1 and Opa1 was found to be involved in the remodelling of mitochondrial networks. The results of western blot showed the expression of Drp1 and Fis1 was upregulated while expression of Mfn1 and Opa‐1 was downregulated after LPS (Figure 4E–I). Yap1 overexpression suppressed the levels of mitochondrial fission‐related proteins (Drp1, Fis1) and restored the expression of fusion‐related factors (Mfn1, Opa1). The cell viability was decreased after LPS, and Yap1 overexpression could improve the cell viability (Figure 4K). These results suggest that Yap1 could improve LPS‐induced mitochondrial dysfunction via modulating mitochondrial dynamic homeostasis.

### 3.5 Yap1 overexpression inhibited neurons ferroptosis in LPS‐induced HT22 cells

**FIGURE 5:**
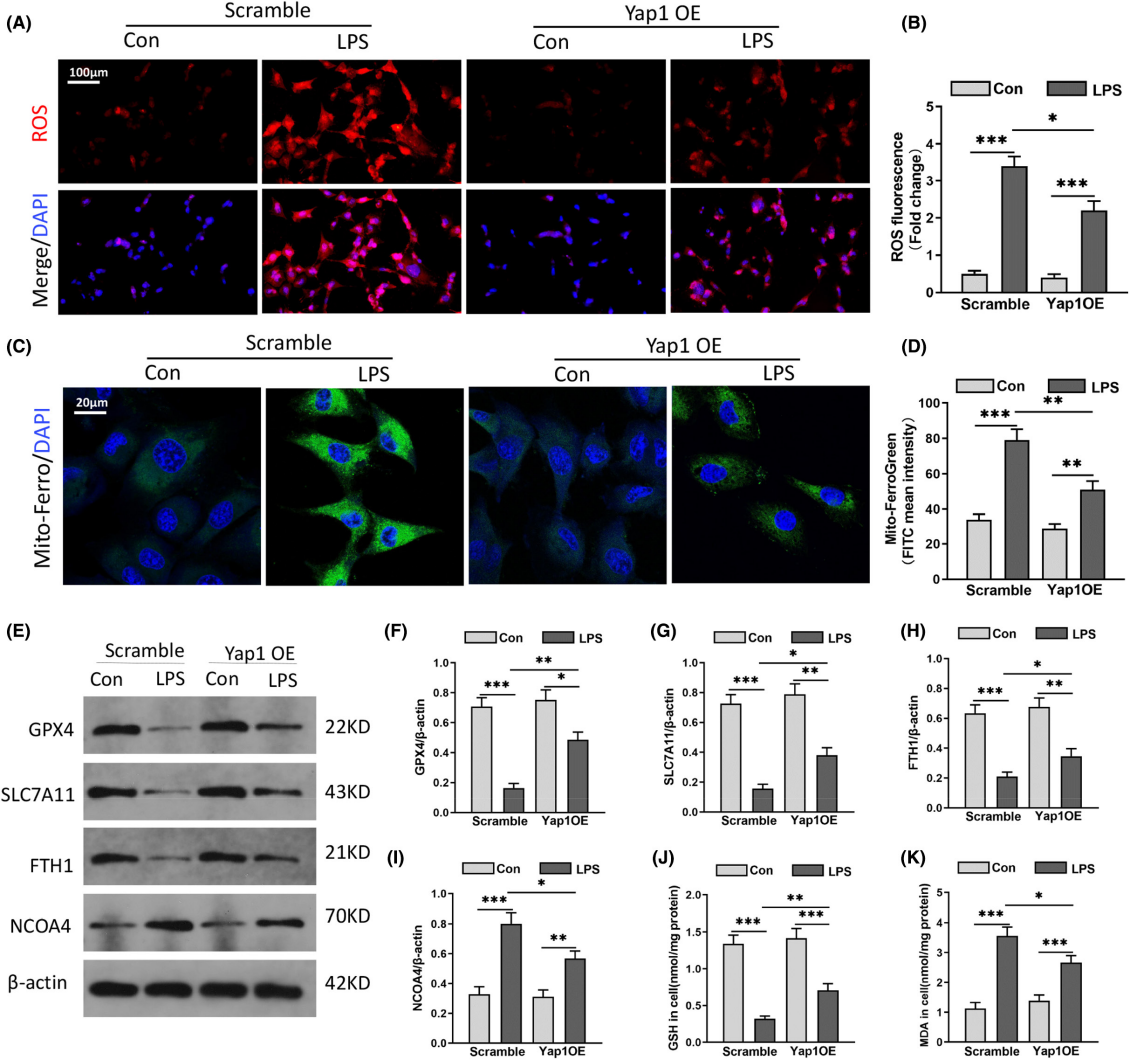
Yes‐associated protein 1 (Yap1) overexpression inhibited neurons ferroptosis in LPS‐induced HT22 cells. (A) Representative images of intracellular ROS production in HT22 cells detected by fluorescence probes. (B) The quantification of ROS fluorescence intensity in each group. (C, D) Mito‐Ferro fluorescence probe was showed by confocal imaging in HT22 cells stimulated with LPS. The images exhibited the localization of ferrous iron in survival cells. The mitochondria stained by Mito‐Tracker Green fluorescence (Scale Bar = 20 μm). (E–I) Western blot and quantification of GPX4, SLC7A11, FTH1 and NCOA4 protein in HT22 cells. (J, K) The commercial kits were used to determine the contents of GSH and MDA of HT22 cells. Data are expressed as mean ± SEM. *p < 0.05, **p < 0.01, ***p < 0.001.

Yap1 overexpression lentivirus (Yap1 OE) was transfected into HT22 cells to explore the relationship between Yap1 and ferroptosis activation in LPS‐induced brain injury. LPS caused significant ROS accumulation in HT22 cells, as evidenced by increased red fluorescence, whereas Yap1 overexpression reduced ROS fluorescence intensity significantly (Figure 5A,B). The Mito‐Ferro fluorescence probe showed that Yap1 could decrease the contents of mitochondrial Fe^2+^ in LPS‐induced HT22 cells, while this effect was partly reversed by Yap1 OE (Figure 5C,D). These results implied that LPS could promote ferroptosis, and this effect could be partly reversed by Yap1 overexpression in vitro.

Yap1 overexpression increased GPX4 levels in the LPS group (Figure 5E,F). FTH1 and NCOA4 are key factors in mitochondria‐related ferroptosis and iron‐related cell death. To understand the influence of Yap1 on ferritinophagy in HT22 cells after LPS, the levels of FTH1, NCOA4 and SLC7A11 were determined by western blot. These results showed that LPS stimulation inhibited the level of anti‐ferroptosis protein SLC7A11 and FTH1, while elevateing the level of pro‐ferroptosis protein NCOA4 in HT22 cells. Therefore, Yap1 overexpression could partly reverse the decrease in SLC7A11 and FTH1 and the increase in NCOA4 (Figure 5E,G–I). Overall, we measured the level of GSH and MDA. After LPS treatment, the content of MDA increased as well as GSH decreased, while Yap1 overexpressing increased the content of GSH and decreased the content of MDA (Figure 5J,K). Summary, Yap1 could prevent ferritinophagy‐mediated ferroptosis in HT22 cells after LPS.

### 3.6 Yap1 overexpression inhibited ferroptosis by disrupting the NCOA4−FTH1 interaction

**FIGURE 6:**
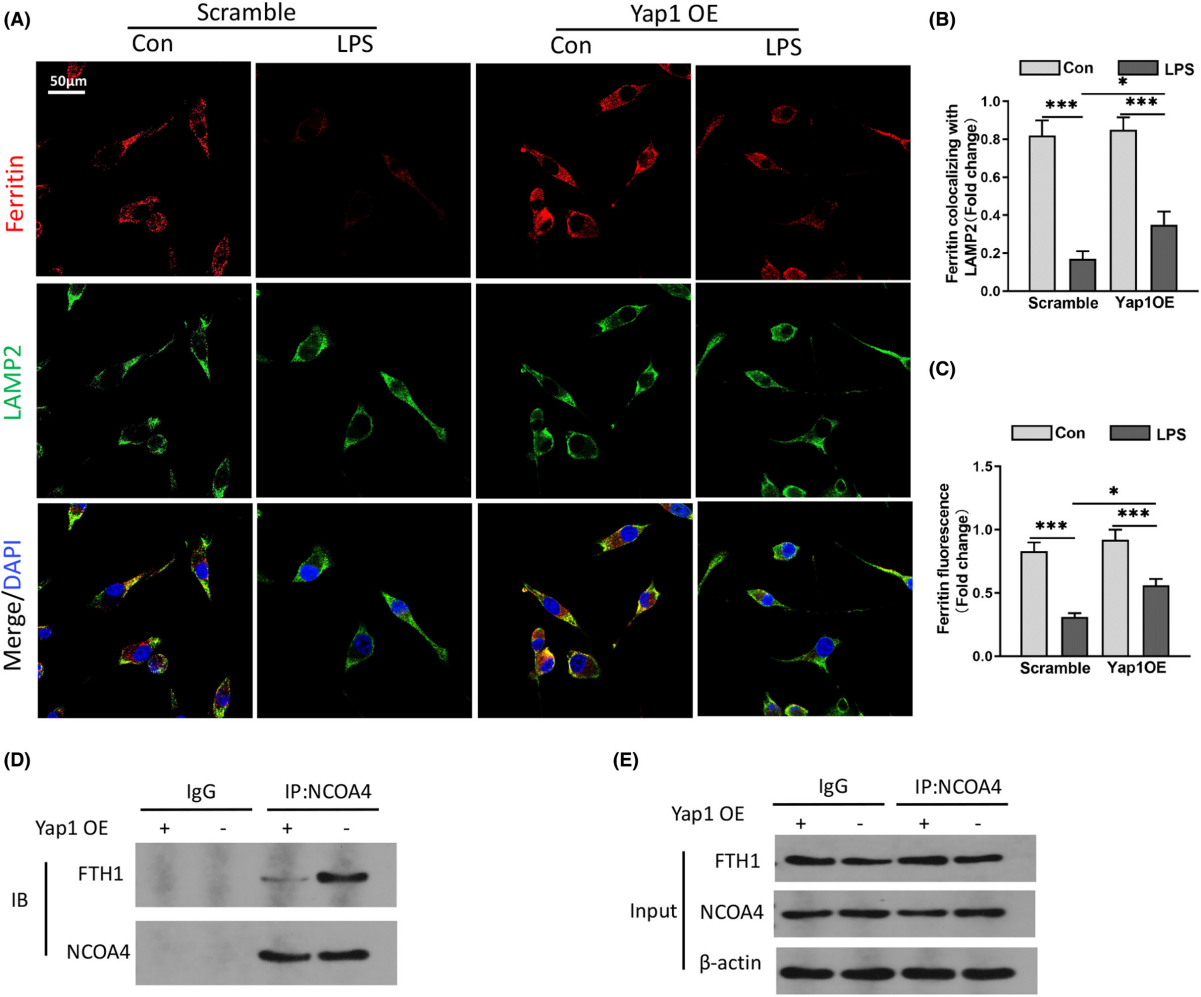
Yes‐associated protein 1 (Yap1) overexpression inhibited ferritinophagy mediated‐ferroptosis by disrupting the NCOA4 − FTH1 interaction. (A) Confocal images of ferritin (red) and LAMP2 (green) were showed in LPS treated HT22 cells, while lysosomes were stained by LAMP2 (green). (B, C) Quantification of the immunofluorescence intensity of ferritin colocalized with LAMP2 and ferritin fluorescence intensity. (D, E) Immunoprecipitation analysis of NCOA4 and FTH1 interaction in HT22 cells. IgG was used for control. Data are expressed as mean ± SEM.*p < 0.05, **p < 0.01, ***p < 0.001.

In mammals, ferritin is the primary iron‐storage protein, It is essential for iron homeostasis and preventing Fenton reaction. As Yap1 overexpression inhibited the contents of Fe^2+^ in mitochondria when compared to the LPS group, we used immunofluorescence assays to detect lysosome location of ferritin to validate the assumption (ferritin is degraded in lysosome through autophagy). Immunostaining of HT22 cells with red fluorescence‐labelled ferritin and green fluorescence‐labelled LAMP2 antibodies showed Yap1 overexpression caused more extensive ferritin fluorescence staining than the scramble group in LPS‐induced cells (Figure 6A–C), suggesting Yap1 definitely inhibited ferritin‐lysosome colocalization and impaired ferritinophagy after LPS (Figure 6A–C).

To further investigate the interaction of NCOA4‐mediated ferritinophagy and FTH1, Co‐IP experiments were carried out to test whether Yap1 inhibits the NCOA4‐FTH1 interaction to prevent ferritinophagy. The results showed that Yap1 overexpression decreased the amount of FTH1 that was bound to NCOA4. This suggests Yap1 could inhibit the interaction between NCOA4 and FTH1 (Figure 6D,E). These results suggested that Yap1 inhibited ferritinophagy‐mediated ferroptosis by interfering with the NCOA4‐FTH1 interaction.

### 3.7 Silencing of Yap1 aggravated neuron ferroptosis in LPS‐induced HT22 cells

HT22 cells were transfected with Yap1 silencing lentivirus (Si‐Yap1) to investigate the effect of Yap1 silence after LPS. After LPS stimulation, the contents of ROS, Fe^2+^and MDA increased and the level of cell viability, GSH and MMP decreased (Figure 6A–F). However, Yap1 silencing further exacerbated the change, the levels of ROS, MDA and Fe^2+^ in mitochondria were increased, while GSH, cell viability and MMP were decreased in HT22 cells (Figure 6A–F). The results suggested that Yap1 silencing could worsen ferroptosis in HT22 cells after LPS‐stimulation.

## 4 DISCUSSION

SAE is a serious consequence during sepsis. It is characterized by diffused neurological dysfunction and cognitive impairment caused by systemic inflammation.^28^ It is the most common encephalopathy in the intensive care unit (ICU), with inattention, agitation, trance, lethargy and even coma presenting in 8%–70% of sepsis patients.^29^ Many studies showed that SAE is associated with significant long‐term cognitive sequelae,^30^, ^31^ and hippocampal neuronal loss in SAE mice was proposed to be the cause of cognitive deficits.^32^

Yap1 is essential for the development of several organs^33^ and is involved in the pathogenesis of many diseases such as cancer, atherosclerosis, ischemia–reperfusion injury, inflammation and others.^34^ By inhibiting TRAF6‐mediated NF‐κB activation, Yap1 can inhibit inflammation and regulate lung endothelial cell activation.^35^ Studies indicate that Yap1 can reduce oxidative stress and apoptosis in the congenital inflammatory response and prevent hepatic ischemia reperfusion injury.^36^ Furthermore, Yap1 could influence astrocyte‐driven microglial activation and negatively regulate neuroinflammation in the nervous system to prevent astrogliosis and affect microglial activation in acute cerebral ischemia–reperfusion injury.^37^

**FIGURE 7:**
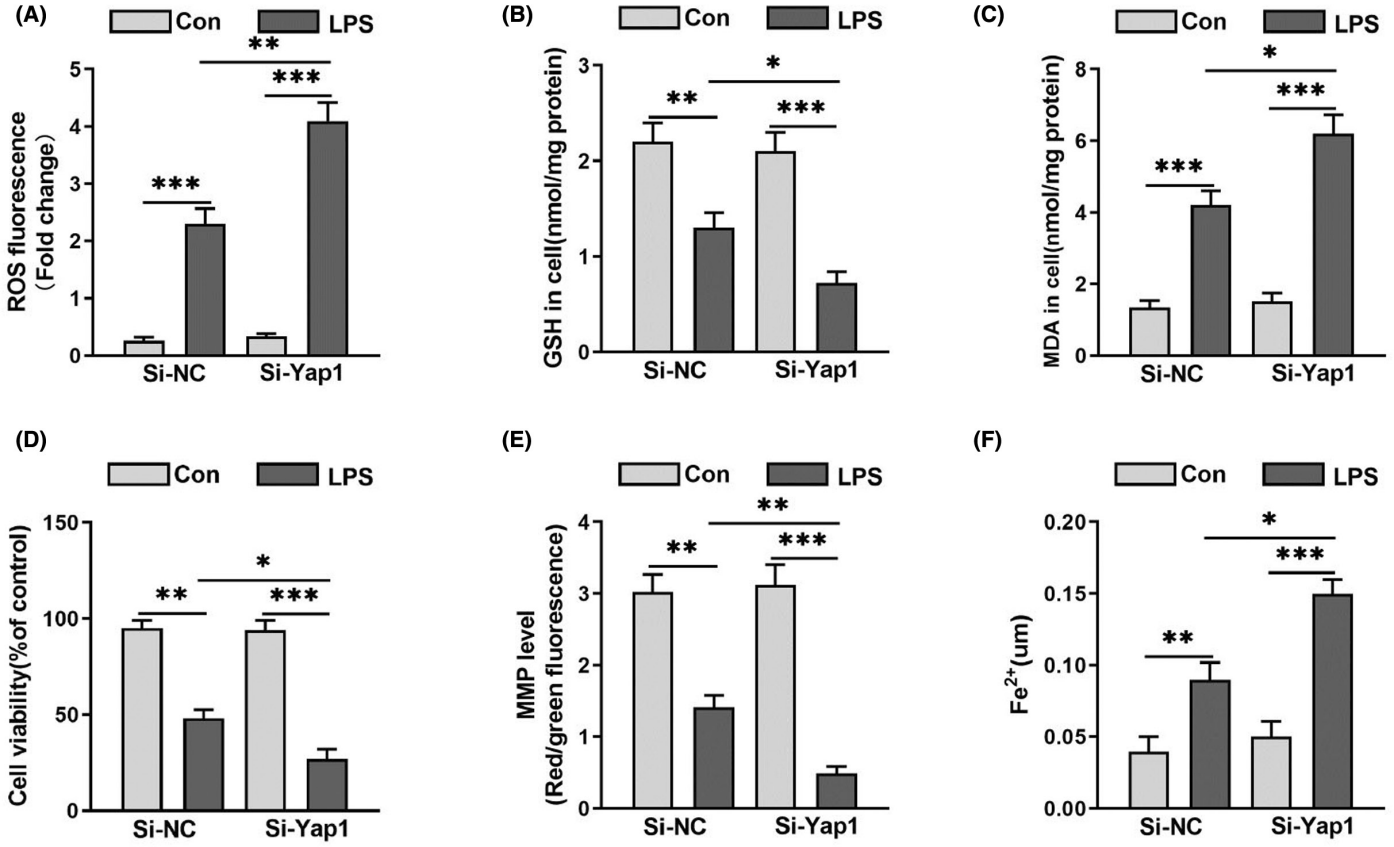
Yes‐associated protein 1 (Yap1) silencing aggravated neurons ferroptosis in HT22 cells following LPS treatment. (A, E) The level of ROS fluorescence intensity and mitochondrial membrane potential (MMP) were detected in HT22 cells. (B, C, F) The contents of GSH, MDA and Fe^2+^ of HT22 cells were determined using the indicated kits. (D) Cell viability was evaluated by the Cell Counting Kit‐8 of HT22 cell. Data are expressed as mean ± SEM. *p < 0.05, **p < 0.01, ***p < 0.001.

Given the positive effect of Yap1 in other diseases, we hypothesized that it could protect against SAE. Yap1 conditional knockout significantly aggravated learning and memory dysfunction, as well as hippocampal pathological damage, inflammatory response, ROS accumulation, mitochondrial fission and Fe^2+^ overload in sepsis mice induced after CLP. Meanwhile, Yap1 overexpression partially reversed the inflammatory response, reduced ROS production, resisted Fe^2+^ overload, and increased mitochondrial fusion. However, the mechanism remains unclear, so we investigated the effect of Yap1 in SAE, which may provide a target strategy to alleviate sepsis‐induced SAE (Figure 7A–F).

Ferroptosis is a type of regulated cell death characterized by iron‐dependent lipid peroxidation accumulation, impaired glutathione metabolism and mitochondrial dysfunction.^38^ The selenoenzyme glutathione peroxidase 4 (Gpx4), which detoxifies membrane lipid hydroperoxides and prevents unchecked toxic lipid peroxidation, is a main endogenous suppressor of ferroptosis. Fe^2+^ participates in numerous biochemical processes in the brain, which has high Fe^2+^ concentrations. Researchers found that ferroptosis is crucial to neurodegeneration at both biochemical and molecular levels, and prevents neuronal death via ferroptosis inhibitors.^16^ Wang's study found that ferroptosis plays a role in the pathogenesis of SAE, and inhibiting ferroptosis protected against SAE by reducing cognitive dysfunction, neurological deficits, disruption of the blood–brain barrier and neuroinflammation, which provided a therapeutic target for SAE.^39^

Yap1 plays key roles in modulating mitochondrial function and ferroptosis in various diseases, and it has been proven to have anti‐ferroptosis effects during the process of ferroptosis in cancer cells.^40^ However, the underlying mechanism for the anti‐ferroptosis effect of Yap1 in SAE is not clear. We explored the role of Yap1 in SAE both in vitro and in vivo. We measured oxidative stress markers, intracellular Fe^2+^ and lipid ROS, and discovered that they were increased in both LPS‐stimulated HT22 cells and the hippocampus of CLP mice. Yap1 conditional knockout further aggravated Fe^2+^ overload and lipid ROS in CLP mice. Yap1 overexpression could reduce the accumulation of Fe^2+^ and lipid ROS in HT22 cells. The levels of GPX4 and SLC7A11 decreased in the hippocampus of CLP mice, while Yap1 knockout further aggravated this change. Microglia are considered to be immune cells in the central nervous system. Previous study showed microglial activation and neuron dysfunction in an animal model of SAE could lead to cognitive impairment.^41^ In our study, Yap1 knockout increased microglia activation and neuroinflammation by increasing the contents of the inflammatory cytokines TNF‐α, IL‐1β and IL‐6, which may be involved in hippocampus lipid peroxidation and increased ferroptosis. These results suggest that Yap1 is involved in ferroptosis by regulating mitochondrial homeostasis in SAE.

**FIGURE 8:**
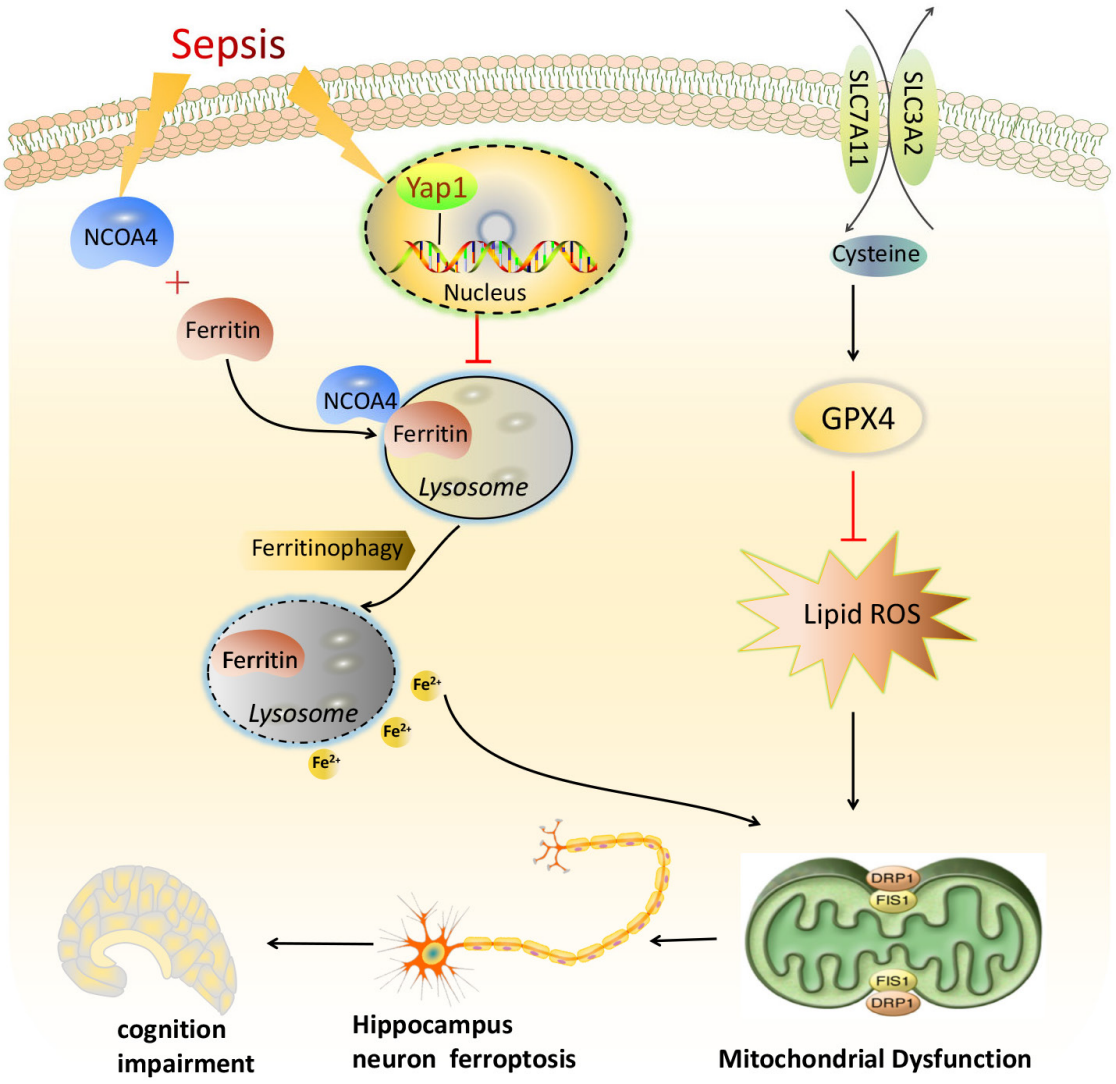
The mechanism illustration for the involvement of Yes‐associated protein 1 (Yap1) in ferritinophagy and ferroptosis in sepsis‐induced brain injury. Sepsis induces increased intracellular NCOA4 and performs NCOA4‐FTH1 interaction. Yap1 can suppress the degradation of ferritin into autophagosomes via inhibiting NCOA4‐mediated ferritinophagy, and prevent ferrous iron from being releasing into mitochondria. Yap1 restrains the accumulation of mitochondrial reactive oxygen species and lipid peroxidation, and conditional Yap1 knockout aggravated mitochondrial fusion and mitotic imbalance in caecal ligation puncture (CLP) mice. Therefore, Yap1 could improve hippocampus mitochondrial function through maintaining mitochondrial dynamic homeostasis, alleviate hippocampus ferroptosis and eventually improve cognitive impairment in sepsis‐associated encephalopathy (SAE).

Mitochondria is considered the main organelle for energy metabolism and the pro‐apoptotic target of reactive oxygen species excess. Mitochondrial fission and fusion are crucial biological events in mitochondrial quality and function. According to previous researches, mitochondrial bioenergetics are disrupted in the hippocampus of mice with Alzheimer's disease, while mitochondrial fission is enhanced and mitochondrial fusion is reduced. It has been reported that Yap1 could regulate mitochondrial fission and fusion.^42^ Yap1 directly participated in inhibiting mitochondrial swelling and the formation of abnormal mitochondria, and reversing collapsed MMP and the damage of the electron transfer chain.^43^ To assess the effect of Yap1 on mitochondrial fission and fusion in SAE mice, the morphological structure of mouse hippocampal mitochondria was observed with transmission electron microscopy. The results showed that conditional Yap1 knockout exacerbated mitochondrial fusion and mitotic imbalance in CLP mice. And the in vitro experiments found that the expression of Drp1 and Fis1 was up‐regulated, while that of Mfn1 and Opa‐1 was down‐regulated, and Yap1 overexpression partly reversed these changes. Thus, our study revealed that Yap1 overexpression improves the learning and memory abilities of SAE mice (Figure 8).

It is reported that ferritinophagy plays a crucial role in ferroptosis.^44^ Ferroptosis can activate autophagy, resulting in ferritin degradation by NCOA4‐FTH1. NCOA4 mediates autophagy of ferritin and degradation of ferritin in the lysosome. Ferritinophagy releases amounts of Fe^2+^ and lipid ROS via Fenton reaction, which eventually initiates ferroptosis. However, NCOA4 deficiency could inhibit the ferroptosis.^45^ A previous study showed that compound 9A reduced Fe^2+^ co‐localization in lysosomes and inhibited the NCOA4‐FTH1 reaction.^46^ Our study showed that Yap1 overexpression reduced the release of Fe^2+^ in lysosomes as well as the degradation of lysosomal ferritin. Furthermore, we discovered that Yap1 can inhibit the NCOA4‐FTH1 interaction via COIP detection. These findings indicate that Yap1 protects against hippocampus neuronal injury and inhibits hippocampus ferroptosis via inhibiting NCOA4‐mediated ferritinophagy. Nevertheless, the direct link between Yap1 and ferroptosis remains unclear. As the signalling pathway of this study is too extensive, further study should be done to demonstrate the role of Yap1 and ferroptosis in SAE.In conclusion, our study demonstrated that sepsis could induce hippocampus ferroptosis via lipid peroxidation and increased iron load, which leads to SAE. Yap1 can improve hippocampus mitochondrial function by maintaining mitochondrial dynamic homeostasis, suppressing hippocampus ferroptosis and eventually alleviating cognition impairment in SAE.

## AUTHOR CONTRIBUTIONS

**Xin Yang:** Investigation (lead); methodology (lead); writing – original draft (lead). **Haifeng Duan:** Conceptualization (equal); funding acquisition (equal); resources (equal). **Sirui Li:** Funding acquisition (equal); investigation (equal); supervision (equal). **Jing Zhang:** Formal analysis (equal); software (equal). **Liang Dong:** Methodology (equal); writing – review and editing (equal). **Jingli Ding:** Methodology (equal); visualization (equal). **Xinyi Li:** Conceptualization (lead); project administration (lead); resources (lead); writing – review and editing (lead).

## FUNDING INFORMATION

This study was supported by Hubei Provincial Natural Science Foundation of China (No. 2021CFB099), Guangxi Natural Science Foundation (Guike AD22035056), the National Natural Science Foundation of China (No. 82472204), Hubei Provincial Natural Science Foundation of China (2023AFB743), the Science, Technology and Innovation Seed Foundation of Zhongnan Hospital (No. CXPY2023042), Translational Medicine and Interdisciplinary Research Joint Fund of Zhongnan Hospital of Wuhan University (Grant No. ZNJC202303).

## CONFLICT OF INTEREST STATEMENT

The authors declare that they have no conflict of interests.

## CONSENT FOR PUBLICATION

Not applicable.

## Data Availability

The datasets used or analysed during the current study are available from the corresponding author on reasonable request.
